# An evaluation of mechanical and biophysical skin parameters at different body locations

**DOI:** 10.1111/srt.13292

**Published:** 2023-02-17

**Authors:** Anto J. U. K. John, Francesco Del Galdo, Rodney Gush, Peter R. Worsley

**Affiliations:** ^1^ School of Health Sciences University of Southampton Southampton UK; ^2^ Raynaud's and Scleroderma Programme, NIHR Biomedical Research Centre Leeds Institute of Rheumatic and Musculoskeletal Medicine, University of Leeds Leeds UK; ^3^ Moor Instruments Axminster UK

**Keywords:** biophysical parameters, functional properties, hydration, mechanical loading, mechanical properties, sensitivity analysis

## Abstract

**Background:**

Skin is the largest organ in the body, representing an important interface to monitor health and disease. However, there is significant variation in skin properties for different ages, genders and body regions due to the differences in the structure and morphology of the skin tissues. This study aimed to evaluate the use of non‐invasive tools to discriminate a range of mechanical and functional skin parameters from different skin sites.

**Materials and methods:**

A cohort of 15 healthy volunteers was recruited following appropriate informed consent. Four well‐established CE‐marked non‐invasive techniques were used to measure four anatomical regions: palm, forearm, sole and lower lumbar L3, using a repeated measures design. Skin parameters included trans‐epidermal water loss (TEWL), pH (acidity), erythema, stratum corneum hydration and stiffness and elasticity using Myoton Pro (skin and muscle probe). Differences between body locations for each parameter and the intra‐rater reliability between days were evaluated by the same operator.

**Results:**

The results indicate that parameters differed significantly between skin sites. For the Myoton skin probe, the sole recorded the highest stiffness value of 1006 N/m (SD ± 179), while the lower lumbar recorded the least value of 484 N/m (SD ± 160). The muscle indenter Myoton probe revealed the palm's highest value of 754 N/m (± 108), and the lower lumbar recorded the least value of 208 N/m (SD ± 44). TEWL values were lowest on the forearm, averaging 11 g/m2/h, and highest on the palm, averaging 41 g/m2/h. Similar skin hydration levels were recorded in three of the four sites, with the main difference being observed in the sole averaging 13 arbitrary units. Erythema values were characterised by a high degree of inter‐subject variation, and no significant differences between sites or sides were observed. The Myoton Pro Skin showed excellent reliability (intra‐class correlation coefficients > 0.70) for all sites with exception of one site right lower back; the Myoton pro muscle probes showed good to poor reliability (0.90–017), the corneometer showed excellent reliability (>0.75) among all the sites tested, and the TEWL showed Good to poor reliability (0.74–0.4) among sites.

**Conclusion:**

The study revealed that using non‐invasive methods, the biophysical properties of skin can be mapped, and significant differences in the mechanical and functional properties of skin were observed. These parameters were reliably recorded between days, providing a basis for their use in assessing and monitoring changes in the skin during health and disease.

## 1 INTRODUCTION

The skin is the largest organ of the body and is often referred to as the window to the body's health. Skin is a complex biological organ with non‐linear viscoelastic properties and is composed of three main layers: the epidermis, the dermis and the hypodermis. The epidermis, the top layer of the skin, consists of the stratum corneum (SC), the transparent layer, the granular layer, the spinous layer and the basal layer. The SC acts as a barrier against pathogens that invade the skin, and preventing uncontrolled water loss.^1^ Knowledge about the structure and function of human skin is of interest for dermatology, cosmetic and healthcare disciplines. Any changes or modifications of the structure can be related to numerous skin‐related conditions, for example, atopic dermatitis (AD), and systemic scleroderma. The skin is also vulnerable to external insults, which can result in chronic wounds, for example pressure ulcers and diabetic foot ulcers. Therefore, measurement of skin properties is essential for diagnosing, monitoring, and developing new therapies.^2^

Bioengineering and dermatological tools have been employed to assess skin health and its response to environmental changes.^3^ These include non‐invasive methods to monitor the barrier function, pH, elasticity, blood flow, structural changes and colour of the skin.^4^ Numerous studies have contributed to the understanding of the skin and provided many tools for diagnosis and treatment of skin conditions like AD. They have also been employed in cosmetic studies to assess a range of non‐invasive techniques based on changes in the electrical characteristics of the skin (such as capacitance, conductance, or impedance) that occur as a function of the skin's water content.^5^, ^6^ Based on capacitance measurements, Corneometer is frequently considered the most accurate instrument for determining the water content in dry situations. However, arbitrary hydration units are used to transform the capacitance measurements of the skin surface.^7^ To discriminate melanin from skin erythema, different technologies have been developed with a variable level of reliability.^8^ Trans‐epidermal water loss (TEWL) is considered one of the most important parameters for skin barrier function. Earlier descriptions for its measurement can be traced back to the 1940s and 1950s. Several TEWL measurement devices with various technologies are currently commercially available and often utilised in routine dermatological assessments and research across the globe.^9^

The thickness of each layer of skin depends on age, body part or skin moisture content.^10^ Mechanical testing of human skin poses major challenges, with many studies limited to ex‐vivo assessment. However, measuring the mechanical properties of human skin can help quantify the effectiveness of dermatological products and identify skin diseases.^11^ Various non‐invasive techniques have been developed for this purpose, with the most common involving suction, torsion and tensile forces.^12^, ^13^, ^14^, ^15^, ^16^ In recent years, there has been further development of non‐invasive tools to evaluate the mechanical properties of soft tissues. For example, the MyotonPro (Myoton SA) is a non‐invasive, hand‐held myotonometer used to assess the viscoelastic properties of soft tissues.^17^ The principle relies on an external short (15 ms), low‐intensity (0.58 N) mechanical impulse applied to the skin. The oscillatory tissue response is then recorded, and subsequent calculations of tissue resting tension, elasticity and stiffness are performed by the internal software using an acceleration graph. This device has shown great reliability for muscle and skin stiffness assessment. Therefore, MyotonPRO can be considered a reliable device for assessing skin stiffness.^18^, ^19^

However, as highlighted in a recent review by the author, data on established bioengineering tools to distinguish between mechanical, chemical and environmental challenges are limited.^3^ Currently, numerous established bodies of literature for Myoton muscle probe at different sites exist. However, this new skin probe introduces tangential load specifically on the skin surface to look at more superficial stuffiness parameters, by implication the tangential load may be more challenging to enact repeatably, because of contact conditions of the skin. Those skin sites with thicker SC and higher density of sweat glands may present a greater challenge than others. There is a need to assess skin stiffness repeatability, which could provide complimentary insight into skin structure and function along with those which are already established in literature namely: TEWL and SC hydration. Therefore, the study aimed to use a combination of biophysical and mechanical skin assessments at distinct skin sites with known structural and functional differences, assessing the between day reliability in a cohort of healthy volunteers.

## 2 MATERIALS AND METHODS

The study involved a repeated measure observational study. Each participant completed the full protocol of multiple measurements at each skin site, in addition to a repeat assessment for intra‐rater reliability evaluation.

### 2.1 Participants

Participants were recruited from the local university population. Exclusion criteria included a history of skin‐related conditions or neurological or vascular pathologies that could affect tissue health. Institutional ethics was granted for the study (ERGO‐65529), and informed consent was obtained from each participant prior to testing. The volunteers without skin diseases and scars, tattoos or wounds in the investigated area took part in this study. Before the measurements were conducted, each participant spent 10 min in the examination room to acclimatise to the environment.

### 2.2 Test equipment

An array of measurement techniques, including biophysical tools and an indenter device, was employed to assess the skin health Table 1.

**TABLE 1:** Description of the parameters analysed

Device	Parameters	Description	Units
Myoton Pro muscle probe Myoton AS, Tallinn, Estonia)	Biomechanical and viscoelastic properties	Axial elasticity (recovery rate after deformation) ^17^, ^18^, ^19^, ^20^	N/m
Myoton Pro Skin Probe Myoton AS, Tallinn, Estonia)	Biomechanical and viscoelastic properties	Tangential elasticity (recovery rate after deformation)^25^, ^26^	N/m
Corneometer CM825 Corneometer MPA9, Courage & Khazaka, Germany	Capacitance	Hydration level of the SC of the epidermis ^14^, ^15^, ^16^	AU
The Mexameter Courage & Khazaka, Germany	By reflectance	A receiver measures the reflection from the skin. As the quantity of emitted light is defined, the quantity of light absorbed by the skin can be calculated^16^.	AU
TEWL, MPA9, Courage & Khazaka, Germany	TEWL	Evaporation rate from the skin ^21^, ^22^, ^23^	g m−2 h−1

Abbreviations: AU, arbitrary units; TEWL, trans‐epidermal water loss.

TEWL was measured according to international guidelines^20^ by placing the probe in gentle contact with the skin for one minute, sampling data at 1 Hz. A mean value of TEWL was estimated over a 5‐s window during a period of equilibrium and recorded in defined units (g m^−2^ h^−1^). Skin hydration was assessed using the Corneometer CM 825, which has been shown to be a reliable tool for the in vivo measurement of skin hydration in terms of sensitivity and reproducibility.^21^ The Skin erythema was measured using a Mexameter (Mx 18 W) a wireless, Conformité Européenne (CE) marked, hand‐held device. Evaluations of skin hydration and erythema were repeated 5 times, exerting a constant pressure onto the skin with the probe held vertically, and the arithmetic mean of 5 values was used for analysis, represented by arbitrary units (AUs).

Soft tissue stiffness was measured using a handheld digital device, MyotonPRO (MyotonAS, Tallinn, Estonia). A standard probe and J‐shaped Skin Probe were placed perpendicular to the skin surface over the target sites, for muscle and skin assessments, respectively. MyotonPRO uses a triaxial accelerometer with the muscle probe held vertical, to perform the mechanical tap.^22^ By contrast, the J‐shape probe applies the impulses horizontally and parallel to the skin surface. To obtain firm contact between the L‐shape probes and skin thin (0.1 mm) double‐sided stickers (10 mm diameter sticker attached to the disc) were used.^18^ For each probe, an initial force was exerted on the skin surface (0.18 N), and an additional mechanical force (0.4 N) for 15 ms, with a quick release, was applied on the skin surface to induce local deformation. An accelerometer with a sampling rate of 3200 Hz was used to record the resultant damped natural oscillations caused by the viscoelastic properties of the tissue.

$$\mathrm{Dynamic}\;\mathrm{stiffness}\left(N/m\right)=\mathrm{amax}\cdot \mathrm{mprobe}/\Delta l,$$

where amax represents the maximum amplitude of the acceleration of oscillation (mG); mprobe represents probe mass, and Δl represents the maximum displacement of the tissue (mm) with a pre‐compression.^23^ The reliabilities and validities to assess the active muscle and skin stiffness have been shown in previous studies.^18^, ^24^, ^25^

### 2.3 Data collection

**FIGURE 1:**
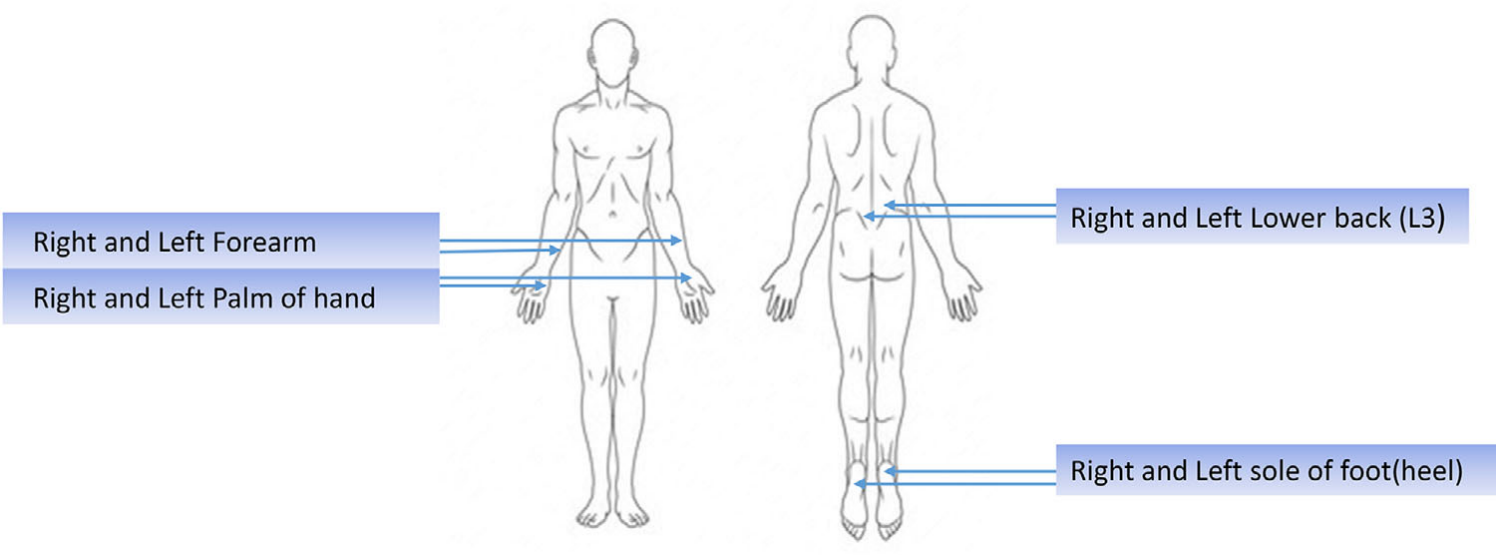
Data collection sites.

All the measurements were carried out in the Biomechanics Testing Laboratory at the Clinical Academic Facility of Southampton General Hospital, with the environment controlled at a temperature of 23 ± 2°C and relative humidity of 42 ± 6%. Participants were requested to wear comfortable loose‐fitting clothing and attend data collection sessions on two consecutive days. Demographic information, including age, height, weight, sex and ethnic background, was collected at the start of the session. Parameter's indicative of the structure and function of skin were assessed in four different sites: forearm, sole of foot (heel) palm (middle) and L3‐lower lumbar, on the right and left sides (Figure 1) using the array of tools. During the session, participants were asked to lie down comfortably in both supine and prone positions to collect al measurements.

### 2.4 Data analysis

All biophysical measurements and MyotonPRO readings were imported into Microsoft Excel. SPSS Statistics v28 was used for data analysis. Data were examined for normality using the Shapiro‐Wilks test and frequency histograms. Subsequently, parametric descriptive statistics (mean, Standard deviation (SD) and range) were calculated bilaterally for each parameter. To test differences between sites, paired *t*‐tests were conducted for each parameter using a confidence interval of 95% (significance of *p* < 0.05). To examine the bivariate associations between the skin output parameters, Pearson's correlation coefficients were performed.

Analysis of reliability was conducted using intra‐class correlation coefficients (ICCs) for each parameter between the two sets of measurements, using an average measures ICC model.^1^, ^3^ The following classification was used for interpreting the level of reliability from ICCs: excellent, >0.75; good to fair = 0.74−0.4; poor, <0.4.^26^ Bland–Altman analysis assessed the variability between the two sets of measurements for each parameter to determine the level of agreement.^27^

## 3 RESULTS

Fifteen (eight males and seven females) healthy volunteers were recruited from the local community. The participants were aged between 25–50 years (mean age 33.13 years) with a mean height and weight of 170 ± 7 cm and 74 ± 15 kg, respectively. They had a corresponding mean body mass index (BMI) of 20.7–32.85 kg/m^2^. The individual skin values at each of the four test sites are presented for the five different biophysical parameters.

### 3.1 MyotonPro skin and muscle probes

The data from the MyotonPro muscle probe revealed a high degree of consistency between right and left sided measurements for three of the four test sites. The only significant difference was observed in the forearm, with right values higher than left (mean difference, 48.73 N/m, 95% Confidence interval (CI) 15.06–82.39, *p* < 0.05) Table 2. By contrast, two out of the four skin probe measurements sites revealed a significant difference (*p* < 0.05) between the right and left sides, corresponding to the sole (mean difference, 88.53 N/m, 95% CI 16.74–160.32, *p* < 0.05) and the forearm (mean difference, 37.00 N/m, 95% CI 5.22−68.77, *p* < 0.05).

**TABLE 2:** Shows the significances between sites

Sites	Side	Myoton muscle probe(N/m) (Mean ± SD)	Significance right versus left *p* values	Myoton skin probe (N/m) (mean ± SD)	Significance right versus left *p* values
Palm of hand	Right	754 ± 108	0.363	773 ± 144	0.663
	Left	759 ± 108		756 ± 164	
Forearm	Right	354 ± 63	0.008^*^	356 ± 153	0.026^*^
	Left	303 ± 60		319 ± 134	
Sole of foot	Right	643 ± 85	0.606	1006 ± 179	0.019^*^
	Left	664 ± 95		917 ± 174	
Lower back/L3	Right	209 ± 41	0.716	482 ± 143	0.881
	Left	208 ± 44		484 ± 160	

**FIGURE 2:**
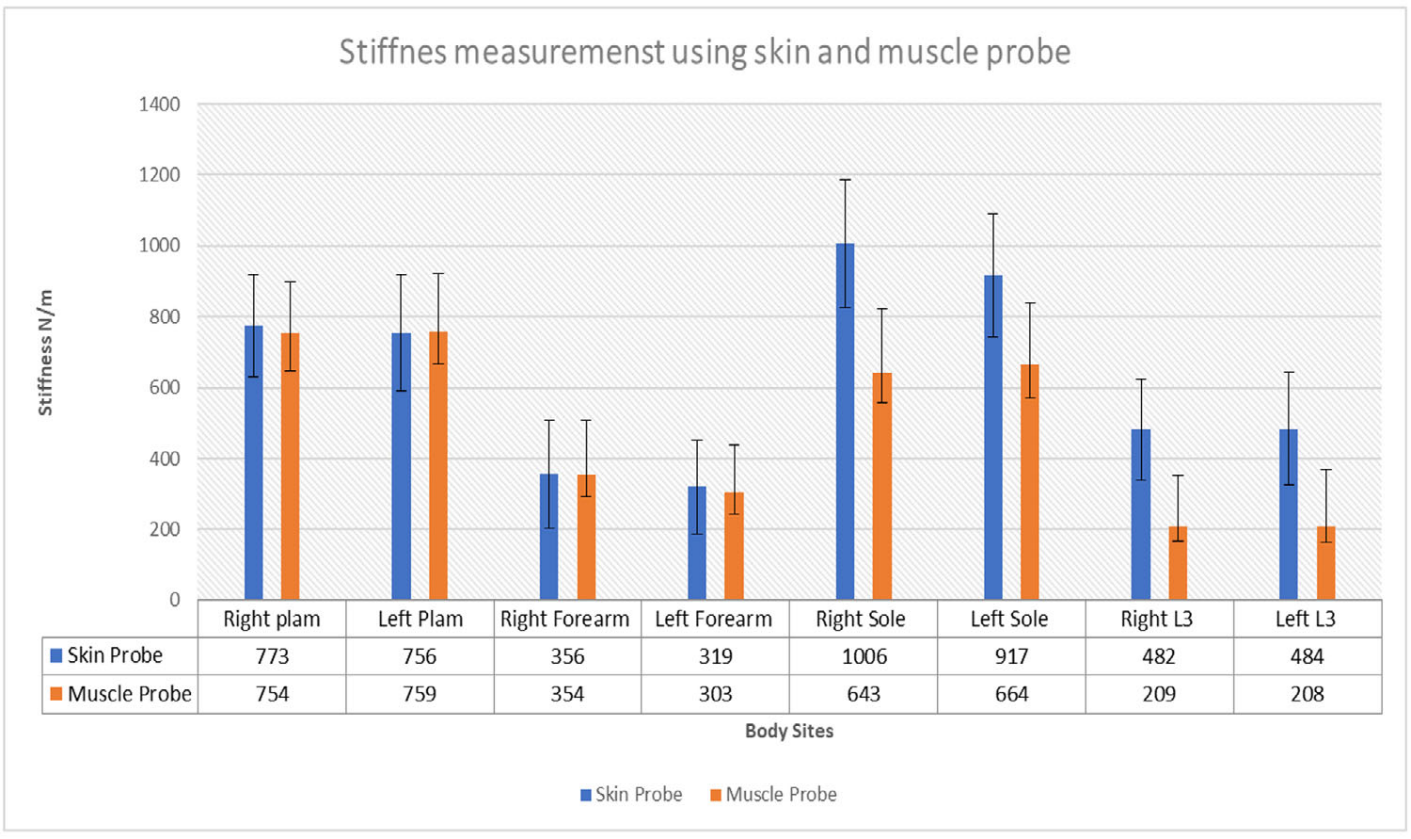
Average stiffness values using skin probe and muscle probe from four different body sites.

When different sites were compared, there was significant difference for both skin and muscle probes. Skin stiffness values differed the most between the sole and forearm (mean difference, 602.81 N/m, 95% CI 536.26–669.36.18, *p* < 0.001) and the least between the sole and palm (mean difference, 124.4 N/m, 95% CI 138.0–35.63, *p* 0.004), which corresponded to the highest values across all sites (Figure 2). A similar trend was also observed with the muscle probe, with significantly high stiffness values in the palm and sole compared to the forearm (mean difference, 320.53 N/m, 95% CI 265.76–375.30, *p* < 0.001) and L3 sites (mean difference, 433.21 N/m, 95% CI 389.24–480.38, *p* < 0.001), respectively.

Tissue stiffness estimates between the two difference probes were equivalent at the palm and forearm sites. By contrast, the sole and L3 revealed the skin probe having significantly higher values than the muscle probe in each of these locations, with mean differences of 287.71 (95% CI 226.78–348.65, *p* < 0.001) and 292.71 (95% CI 242.07–343.35, *p* < 0.001), respectively.

### 3.2 TEWL

**FIGURE 3:**
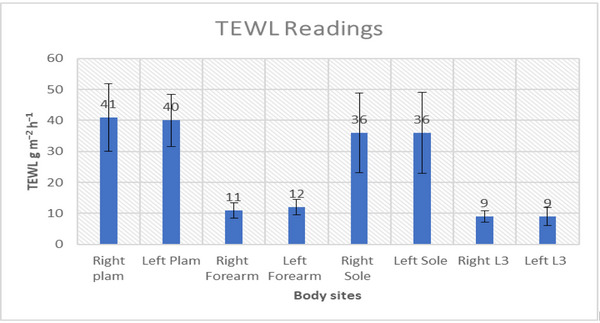
Average values of trans‐epidermal water loss (TEWL) at different body sites.

There were no significant differences (*p* > 0.05) between right and left sided measurements for the TEWL values. There were, however, distinct differences between the different body sites, with the palm and the sole values being three times higher than those of the forearm and L3 sites. In addition to the higher mean values, the palm and sole also revealed the highest degree of inter‐subject variability, with values ranging from 23 to 58 g/m^2^/hr and 12 to 69 g/m^2^/hr for palm and sole, respectively. There was much less variation in the forearm and L3 sites, as indicated by the standard deviation (Figure 3).

### 3.3 Corneometer CM 825

**FIGURE 4:**
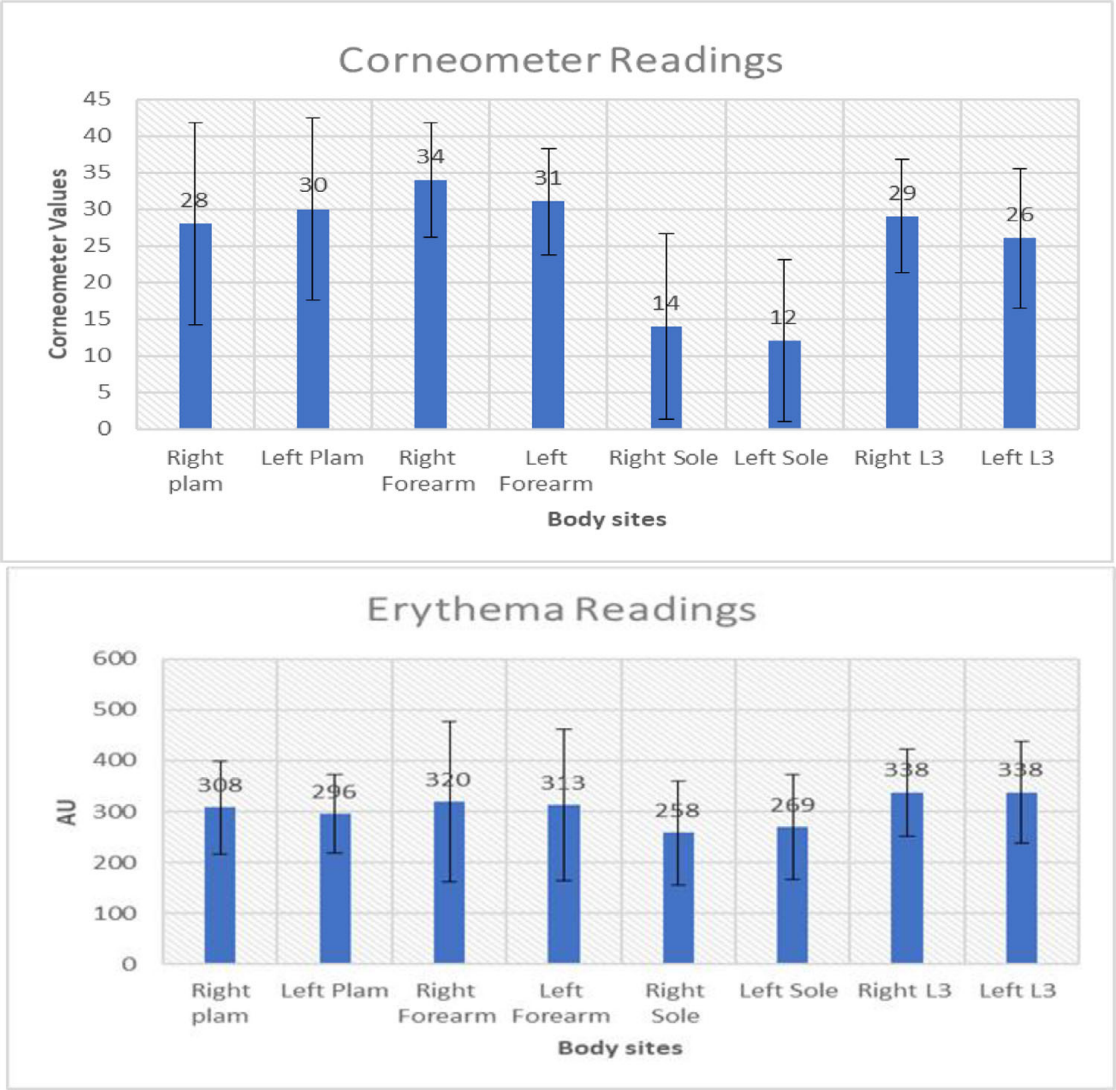
(A) Average Corneometer values recorded from four different body sites. (B) Average erythema values from four different body sites.

Similar skin hydration levels were recorded in three of the four sites, with the main difference being observed in the sole. Indeed, the sole was on average more than 50% lower than the other sites (Figure 4A). In addition to being the lowest recorded site, the sole was also the only site with significant differences between right and left values (mean difference, 1.71, 95% CI 0.2.74–3.19, *p* < 0.05). In a similar trend to TEWL values, hydration levels of the palm and sole of foot revealed a high degree of inter‐subject variation.

### 3.4 Mexameter

Erythema values estimated by the Mexameter were characterised by a high degree of inter‐subject variation and no significant differences between sites or sides were observed (*p* > 0.05). The largest variation was observed in the forearm with values ranging from 98–680 AU. There was a trend for lower values in the sole than in the other three sites; however, due to the high degree of variability in the values, no significant difference was observed (Figure 4B).

### 3.5 Reliability

Across all the skin parameters, the myoton muscle probe, skin probe and Corneometer revealed the highest intra‐rater reliability, with ICC values exceeding the 0.75 threshold indicative of good reliability in most skin sites. By contrast, the TEWL and Mexameter failed to reach this criterion in all test sites with moderate to low reliability observed. There were distinct site‐specific trends in the reliability data, with right L3 having consistently low values across all skin parameters Table 3.

**TABLE 3:** Reliability for all parameters

Sites	Myoton muscle probe ICC (95% CI)	Myoton skin probe ICC (95% CI)	TEWL ICC (95% CI)	Corneometer ICC (95% CI)	Mexameter ICC (95% CI)
Right palm	0.9** (0.71‐0.97)	0.77* (0.34‐0.92)	0.38 (−0.76‐0.79)	0.92** (0.79‐ 0.98)	−0.23 (−3.41‐0.61)
Left palm	0.62* (−0.16‐0.88)	0.73* (0.21‐0.91)	0.59 (−0.20‐0.86)	0.92** (0.77‐0.97)	−1.15 (−7.05‐0.32)
Right forearm	0.48 (−0.42‐0.82)	0.75* (0.29‐0.92)	0.65* (0.05‐0.88)	0.81* (0.45‐0.94)	0.65* (−0.09 −0.89)
Left forearm	0.82* (0.48‐0.94)	0.91** (0.75‐0.97)	0.63* (0.01‐0.87)	0.77* (0.37‐0.92)	0.68* (0.03‐0.90)
Right sole	0.86** (0.60‐0.96)	0.71* (0.07‐0.91)	0.82** (0.50‐0.94)	0.92** (0.79‐0.98)	0.65* (−0‐.07‐0.89)
Left sole	0.62* (−0.04‐0.87)	0.7* (0.16‐0.90)	0.74* (0.24‐0.92)	0.96** (0.89‐0.99)	0.27 (−1.41‐0.76)
Right L3	0.17 (−1.38‐0.72)	−0.15 (−2.77‐0.62)	0.46 (−0.36‐0.81)	0.74* (0.23‐0.91)	0.53 (−0.44‐0.85)
Left L3	0.92** (0.76‐0.97)	0.79* (0.39‐0.93)	0.71* (0.17‐0.90)	0.83* (0.50‐0.94)	0.71* (0.12‐0.91)

Abbreviations: ICCs, intra‐class correlation coefficients; TEWL, trans‐epidermal water loss.

* *p* < 0.05.

** *p* < 0.001.

### 3.6 Bland–Altman analysis

**FIGURE 5:**
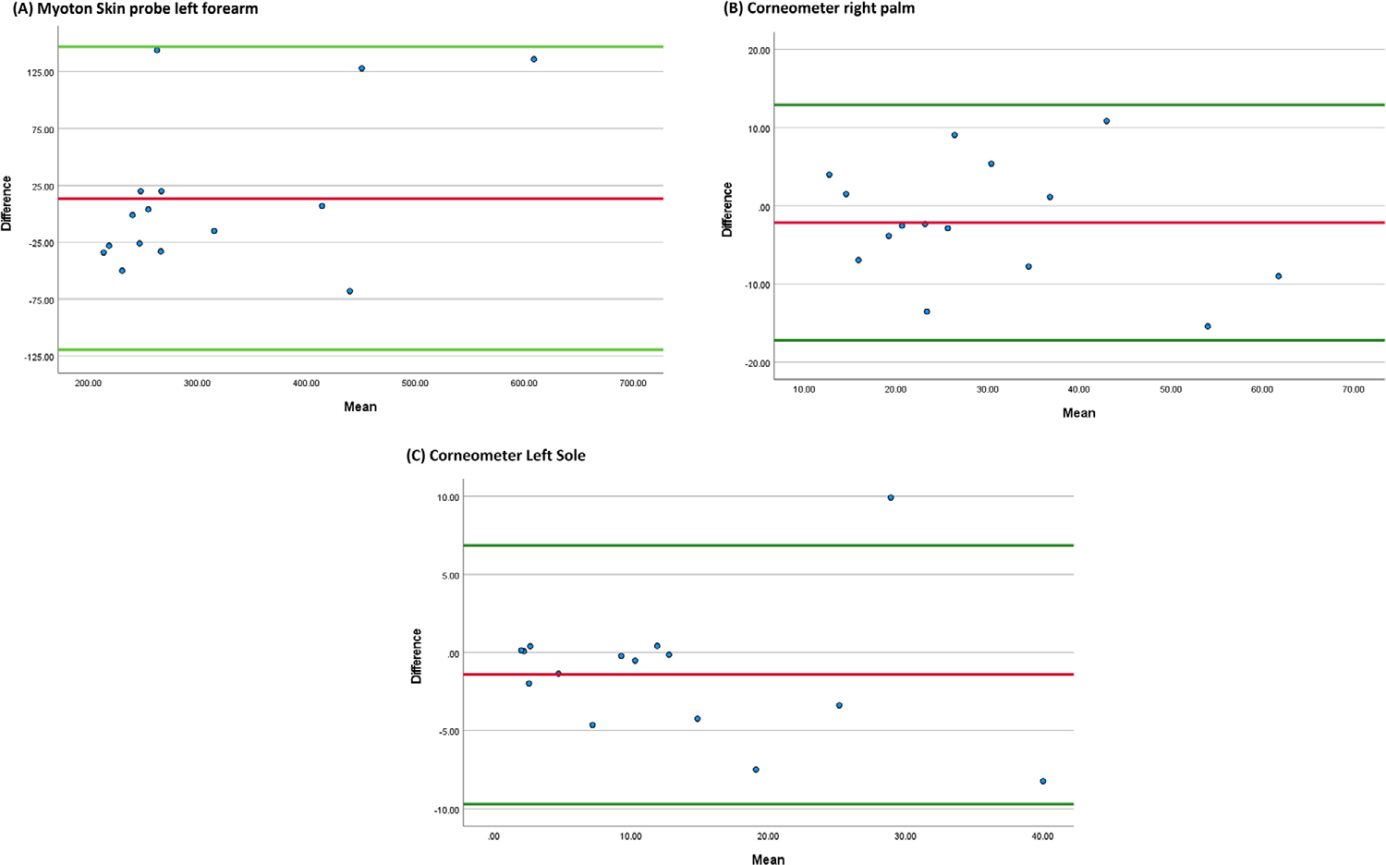
Bland and Altman plots highlighting the intra reliability agreement for (A) Myoton skin probe Left forearm, (B) Corneometer right palm (C) Corneometer left sole. The red line represents the mean difference. The 95% upper and lower limits of agreement are represented by the green line and show two standard deviations above and below the mean difference, respectively.

Bland and Altman plots revealed there was no significant difference between the two readings, and good agreement between the measurements. There is no proportional bias in the distribution of data around the mean difference line. There is an agreement, the values in Figure 5A,B are clustered around the mean of the differences, and certainly within 2 standard deviations of the mean. In the Figure 5C the vast majority of the points were within 2 standard deviations bar one participant.

## 4 DISCUSSION

In our study, a comprehensive analysis of variations in different biophysical skin parameters was completed for four different sites, using non‐invasive biophysical and biomechanical tools. Analysis revealed that specific skin parameters were sensitive to detect regional differences in the skin structure and function, namely Myoton (skin and muscle probe) and TEWL. By contrast, skin hydration and erythema were not distinguished between skin sites. Equivalent reliability was demonstrated between Myoton Probes, TEWL and Corneometer, with exception being Mexameter, which was found to be unreliable across sites.

### 4.1 Myoton Pro skin and muscle probe

In the present study, a clear distinction between the sites among all the tested volunteers using skin and muscle probes was identified. The palm and sole were the stiffest as compared to the forearm and lower back which were softest. The measurement on the palm and forearm were similar with both the probes. However, the sole and the lower back measurements were very different between the probes due to the skin structure at the site of measurements. Males had a higher stiffness as compared to females^28^ on the four sites tested when measured using a skin probe. But, there was no significant difference between the genders using the muscle probe. The Myoton Probe data corroborate with our underlying knowledge of the structure and function of these skin sites, where load‐bearing skin sites by implication need a higher degree of stiffness because they have a higher density of collagen and much thicker stratum corneum to tolerate the mechanical loads of daily activities.^29^ The between day reliability of the skin MyoTonPRO probe (ICC 0.70−0.91) and muscle probe (ICC 0.49–0.9) was moderate to high with the exception of the Right lower back (CC value of −0.16‐0.18). These variations are due to skin structure and hair on the sites of measurement, which could have interfered with probe measurements. Change in the orientation of probe namely J shaped/skin probe provides a tangential force against the skin surface; this provides a localised area of displacement and therefore assessment of elasticity of skin. However, this presents the challenge of maintaining secure contact in order to get repeated measurements.

It has been reported that the MyotonPRO is a reliable method for evaluating the mechanical properties of muscles and tendons. The findings of the present study are in agreement with the results of previous studies. The MyotonPRO showed good intra‐ and inter‐rater reliability for the stiffness for both muscle and skin Probe. In one study it was shown that MyotonPRO equipped with J‐shape probes is perfectly suited for measuring skin stiffness in humans.^18^ A previously reported that inter‐observer and intra‐observer ICC values showed great or excellent reliability of the MyotonPRO for stiffness measurement^30^ as well as all other parameters.^19^

### 4.2 TEWL

Even though people have assessed TEWL in different body locations, a number of studies have negated sites with vulnerability such as plantar tissue of the foot but also the body sites where there are evident changes in skin structure and function. However, there is little in the literature on determining site‐specific differences between these parameters and on the ability to reliably assess skin‐specific mechanics. The palm and sole are exposed to the environment and showed higher values for TEWL in all the participants and both genders in our study. The foot and palm have a higher degree of trans‐epidermal water loss that can be attributed to higher density of sweat glands in these regions. There is equality between right and left side measurements in all the measured sites, with no significance found between the sides. Our results corroborate previous published research. The TEWL reading was higher in males in the palm and forearm as compared to Females. The forearm values in our study are consistent with one of the studies.^31^ Higher values in males correlate with their outdoor working habits. Other studies also noticed higher values in males.^32^ One of the studies observed that TEWL in males is lower than in females up to 50 years of age, after which there is no difference.^33^ Numerous other studies did not observe much difference in TEWL between genders.^34^ Ageing plays a crucial role in skin barrier function and is widely accepted but has not yet been conclusively evaluated.^35^, ^36^, ^37^ The intra‐rater reliability of the TEWL ranged from ICC=0.38–0.82 in our study, these values were lower than those cited in other studies where values ranged from 0.86–0.88. This may have been due to the different anaomtical locations under investigation.^38^, ^39^

### 4.3 Hydration

SC hydration plays a vital role in skin function such as regulation of epidermal proliferation, differentiation and inflammation. The hydration values were high on the forearm and back as compared to the palm and sole. In our study, we observed slightly higher hydration in females on the palm and sole as compared to males. Other studies also have reported a similar observation.^32^, ^33^ Some studies have observed no gender differences in hydration, while some have reported no correlation between age with hydration.^34^, ^35^ The between day reliability of corneometer values was high (ICC 0.74−0.963), corresponding to those published in other studies^39^ Table 2.

### 4.4 Mexameter

The erythema scores were found to be highest at the Lumbar L3 body site with an average score of 337.6 AU for the right and 338.4 for the left side. Our data are in contrast to another study published by Nedelec et al.^40^ We found out that skin erythema was higher in females than males on body sites measured but marginally lesser in the left palm and left sole. This was contrary to the findings of Firooz, et al.,^32^ this study also found the leg had the lowest skin erythema, this pattern was also observed in our study(sole).

### 4.5 Limitations

One of the limitations of the study is the results of the parameters are conflicting, which might be due to differences in study design, measurement devices, sample size, measuring site, environmental conditions, and the genetic backgrounds of the subjects. Moreover, the study is conducted on a healthy cohort and further testing on specific groups is critical to translate the findings to a clinical setting. Measurements such as erythema are highly influenced by variations in skin tone associated with individuals of different ethnicity. These differences should be considered when designing clinical studies. Skin hydration and trans epidermal water loss reflect skin barrier function These are influenced by variations in the thickness of the stratum corneum, sebum secretion, cutaneous perfusion, core body temperature, skin blood flow, environmental conditions and many other factors.^41^

### 4.6 Clinical implication

Certain illnesses, such as scleroderma, have site‐specific changes; the changes are more peripheral compared to more central locations; the non‐invasive technologies investigated may be used to characterise skin sites on these patients to record changes and aid in disease monitoring.

## 5 CONCLUSIONS

This study examined baseline values measured at four body sites on the right and left sides, using well‐reported, CE‐marked commercial devices, and reliability was also evaluated. The study showed marked differences in mean baseline values between different sites of the body. Out of all the skin parameters, the myoton muscle probe, skin probe and Corneometer were found to be reliable in identifying differences in most skin sites. On the other hand, TEWL and Mexameter proved less reliable. The study revealed that by using non‐invasive methods, the biophysical properties of the skin can be mapped, and normal ranges can be generated for healthy volunteers. On this basis, skin health could be assessed using these devices in future studies in patients with skin conditions including scleroderma and atopic dermatitis.

## FUNDING INFORMATION

SRUK‐ SP1

## CONFLICT OF INTEREST STATEMENT

The authors wish to confirm that there is no known conflict of interest associated with this publication, and there has been no significant financial support for this work that could have influenced its outcome.

## Data Availability

The data that support the findings of this study are openly available in University of Southampton, Library at https://doi.org/10.5258/SOTON/D2316, reference number: 10.5258/SOTON/D2316.
